# Towards enhancing the representativeness of patient-reported outcomes collection at community cancer centers^☆^

**DOI:** 10.1016/j.apro.2025.100301

**Published:** 2025-12-08

**Authors:** Chengbo Zeng, Neil E. Martin, Peter M. Meyers, Marc DeAngelo, Tanja Xing-Yu He, Yu-Jen Chen, Boyu Ren, Andrea L. Pusic, Maria O. Edelen, Jason B. Liu

**Affiliations:** a Patient Reported Outcomes, Value and Experience (PROVE) Center, Department of Surgery, Brigham and Women’s Hospital, Boston, MA, USA; b Harvard Medical School, Boston, MA, USA; c Department of Radiation Oncology, Brigham and Women’s Hospital and Dana-Farber Cancer Institute, Boston, MA, USA; d Office of the Chief Medical Officer, Mass General Brigham, Boston, MA, USA; e Center for Surgery and Public Health, Department of Surgery, Brigham and Women’s Hospital, Boston, MA, USA; f McLean Hospital, Belmont, MA, USA; g Division of Plastic and Reconstructive Surgery, Department of Surgery, Brigham and Women’s Hospital, Boston, MA, USA; h Behavioral and Policy Sciences, RAND Corporation, 20 Park Plaza #910, Boston, MA, USA; i Division of Surgical Oncology, Department of Surgery, Brigham and Women’s Hospital, Boston, MA, USA

**Keywords:** Radiation Oncology, Community cancer centers, Patient-reported outcome measures, Nonresponse, Representativeness

## Abstract

**Objectives:**

While patient-reported outcome measures (PROMs) can enhance cancer care, their implementation in community-based settings remains underexplored. This study compared patient characteristics and response rates between community and academic hospitals within the Mass General Brigham (MGB) health system, a large integrated health network with a standardized PROM program.

**Methods:**

We analyzed data from 31,164 adult cancer patients seen at three community and two academic radiation oncology clinics within MGB from 2020 to 2024. The Patient-Reported Outcomes Measurement Information System Global-10 health questionnaire was consistently administered to all patients seen at these clinics. Patient characteristics and response rates were compared by site of care.

**Results:**

Community sites had higher proportions of older adults, females, English speakers, and patients with a high school or less education. Over time, PROM collection improved significantly overall and across subgroups at both community and academic sites. Response rates were relatively lower at community sites than at academic sites, with disparities most evident among older adults, males, and those with federal insurance.

**Conclusion:**

Although PROM collection improved over time across MGB-affiliated radiation oncology clinics, older male patients and those with federal insurance remain key targets for further efforts to enhance PROM representativeness between community and academic settings.

## Introduction

Community hospitals provide essential multidisciplinary care for about 80 % newly diagnosed cancer patients, up to 50 % of whom receive radiation therapy [1–6]. While the use of patient-reported outcomes (PROs) has been shown to improve cancer care at large, well-resourced academic hospitals [7–9], their implementation and impact in community settings is underexplored. Given differences in patient demographics, resources, and infrastructure [1,4,9–16], integrating patient-reported outcome measures (PROMs) into routine care at community hospitals may pose unique challenges compared to academic hospitals, potentially hindering equitable access to the benefits of PROMs, particularly for underserved populations [17,18].

We sought to compare patient characteristics and response rates between community and academic hospitals within the Mass General Brigham (MGB) health system, a large integrated health network with a standardized PROM program. To optimize generalizability across cancer types, we used data from routine radiation oncology care, as radiation therapy is a fundamental component of treatment for many cancer types, whether for curative or palliative purposes. Furthermore, to ensure sufficient sample size for subgroup comparisons and to capture meaningful changes in response rates over time, we focused on the annual number of patient encounters at each site of care.

## Methods

### Study setting, population, and data resource

We included adult cancer patients who presented to radiation oncology clinics within the MGB system from 2020 to 2024. In 2012, MGB established a systemwide standardized PROM program, as described in detail elsewhere [9,19–21]. Participation in the PROM program by clinics and completion of PROMs by patients are both voluntary. MGB Radiation Oncology practices uniformly implemented PROM collection for each clinic encounter across all sites in 2015. The Patient-Reported Outcomes Measurement Information System Global-10 health questionnaire (Global-10) version 1.0/1.1 is administered to all patients seen at these clinics [9]. To ensure comparability across sites and over time, we included only clinics that collected the Global-10 consistently throughout the study period. Of the seven Radiation Oncology clinics in the system, five met this criterion, three of which were at community sites and two were at academic sites.

### Response rate

A patient was considered a responder if they completed at least one assigned Global-10 across all assigned time points within a single year. The response rate was defined as the proportion of responders within the study population. We estimated the within-site response rates using observed proportions and reported these estimates, along with their corresponding 95 % confidence intervals (CIs), for the overall cohort and for patient subgroups.

### Statistical analysis

We summarized within-site demographic and clinical characteristics from 2020 to 2024. We used Chi-square tests to compare patient characteristics between academic and community sites by year.

We examined trends in within-site response rates using Cochran-Armitage tests for the overall population and by patient subgroups. Using Chi-square tests, we compared response rates between sites for the overall cohorts, as well as within-site response rates across subgroups. We specifically focused on age group(≤64 years or > 64 years), sex(male or female), race/ethnicity(white or non-white), insurance type(federal or commercial), highest education level(high school or less or college or above), and degree of multimorbidity as measured using Charlson Comorbidity Index(CCI; CCI≤1 or CCI≥2), all of these are common predictors of nonresponse in PROM collection used in prior research [9,22].

In secondary analyses, we examined between-site differences in response rates among subgroups known to have relatively lower response rates, including older adults, males, racial/ethnic minorities, patients with federal insurance, high school or less education, and those with more comorbidities. All analyses were conducted using SAS version 9.4(SAS Institute, Inc.). To account for the false positives resulting from multiple comparisons of response rate across patient subgroups within and between sites, we used Benjamini-Hochberg adjustment.

## Results

### Overview

Among the 31,164 patients across five clinics, 16,939(54 %) only visited academic sites, 12,938(42 %) only visited community sites, and 1287(4 %) visited both. Breast, prostate, and lung cancers were the most common cancer types treated at both sites.

### Between-site differences in patient characteristics

Compared with academic sites, community sites had higher proportions of older adults, females, English speakers, and patients with a high school education or less, but a lower proportion of employed patients (Table 1). Community sites also had more patients with missing data on race/ethnicity, education level, and employment status. In addition, patients treated at community sites had fewer comorbidities.

### Between-site differences in response rates over time

**Fig. 1.**
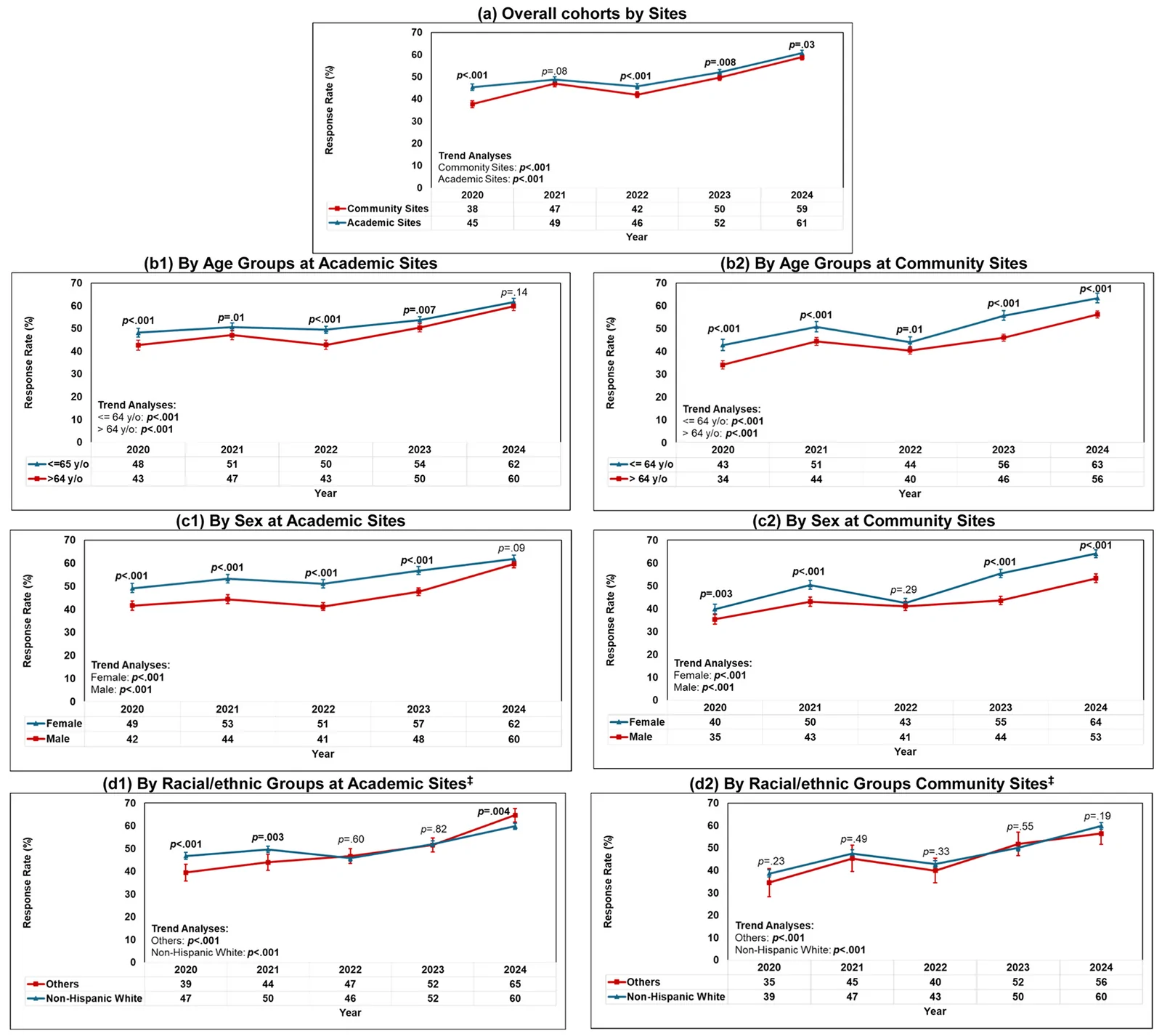

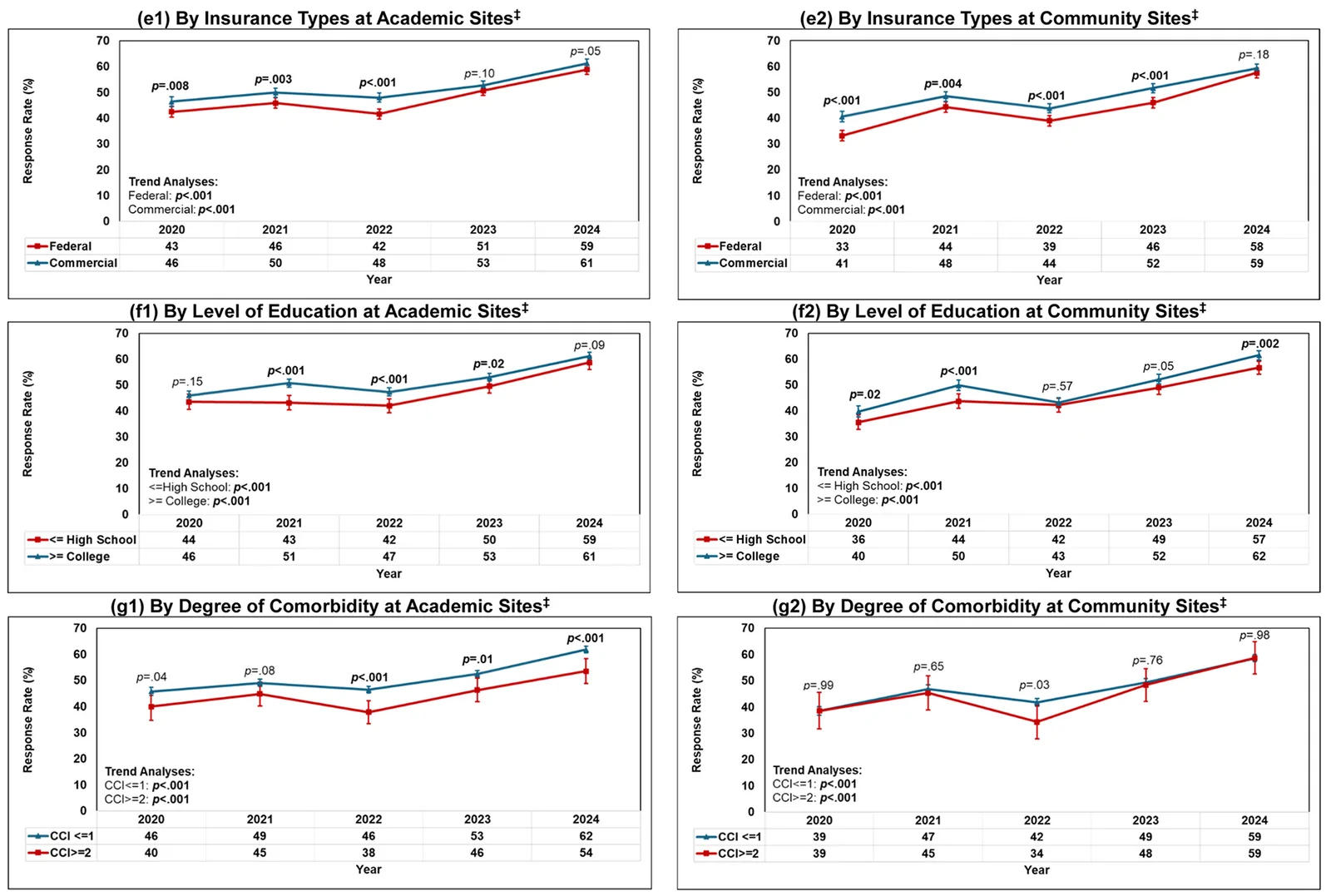
Within-site response rates for the study cohorts and across patient subgroups, 2020–2024. ‡: patients who were missing race/ethnicity, insurance types, education level, or Charlson comorbidity index were excluded. *: *p*-value in bold was statistically significant for the Chi-square test by sites, or for the tests across demographic and clinical characteristics based on the Benjamini-Hochberg adjustment. Interpretation: We examined trends in within-site response rates using Cochran-Armitage tests for the overall population and by patient subgroups. Using Chi-square tests, we compared response rates between sites for the overall cohorts (Fig. 1a), as well as within-site response rates across subgroups (Figs. 1b – 1g). For example, in Fig. 1a, response rates at both sites were increasing from 2020 to 2024. Response rates were consistently lower at community sites compared with academic sites from 2020 to 2024. Overall, the difference between two settings decreased over time, with the difference decreasing from 7 % in 2020–2 % in 2024.

Response rates at both sites were increasing from 2020 to 2024(community sites: 38 %[95 %CI: 36–39 %] to 59 %[95 %CI: 58–60 %], Cochran-Armitage test: *p* < .001; academic sites: 45 %[95 %CI: 44–47 %] to 61 %[95 %CI: 60–62 %], Cochran-Armitage test: *p* < .001; Fig. 1a).

Response rates were consistently lower at community sites compared with academic sites from 2020(community vs. academic sites: 38 %[95 %CI: 36–39 %] vs. 45 %[95 %CI: 44–47 %], *p* < .001) to 2024(community vs. academic sites: 59 %[95 %CI: 58–60 %] vs. 61 %[95 %CI: 60–62 %], *p* = .03). Overall, the difference between two settings decreased over time, with the difference decreasing from 7 % in 2020–2 % in 2024 (Fig. 1a).

### Within-site response rates across patient subgroups

Response rates significantly increased across demographic and patient subgroups from 2020 to 2024(Figs. 1b to 1g, Cochran-Armitage test: *p* < .001). Lower response rates were consistently found among older adults(Fig. 1.b1; Fig. 1.b2), males(Fig. 1.c1; Fig. 1.c2), patients with federal insurance(Fig. 1.e1; Fig. 1.e2), lower levels of education (Fig. 1.f1; Fig. 1.f2), and those with more comorbidities(CCI≥2; only at academic sites[Fig. 1.g1] but not at community sites [Fig. 1.g2]), from 2020 to 2024. For comparisons across racial/ethnic groups, at academic sites, racial/ethnic minorities had significantly lower response rates than the non-Hispanic White population in 2020, 2021, and 2024(Fig. 1.d1). Such differences were not found at community sites (Fig. 1.d2).

### Secondary analyses

Supplemental Figure S1 presents between-site comparisons of response rates among older adults(Figure S1.a), males(Figure S1.b), racial/ethnic minorities(Figure S1.c), patients with federal insurance (Figure S1.d), high school or less education(Figure S1.e), and those with more comorbidities(CCI≥2; Figure S1.f). Notably, there were consistently large differences in response rates between community and academic sites among older adults, from 2020 to 2024. Among males, significant differences were observed in 2020, diminished in 2021 and 2022, but widened again since 2023. Response rates among patients with federal insurance were consistently lower at community sites compared to academic sites, with statistically significant differences in both 2020 and 2023. In contrast, between-site differences among the other patient subgroups were relatively small.

## Discussion

Our multi-year analysis found significant improvements in PROM collection overall and across patient subgroups at both community and academic hospitals from 2020 to 2024. These gains are likely attributable to advances in the electronic health record (EHR) system functionality, successful integration of PROM collection into clinical workflows, and the use of various outreach strategies, including text messaging, embedding PROMs in the electronic pre-visit check-in process, and including PROM links in appointment reminder emails [20,21].

Males and underserved populations, such as older adults, racial/ethnic minorities, patients in low SES, and those with more comorbidities, had lower response rates than their counterparts. These findings suggest that, to improve PROM collection, enhance representativeness, and ensure equitable access to their benefits, outreach strategies and implementation efforts should be tailored to meet the needs of these populations [18,22,23]. While we observed that the gap in response rates between community and academic hospitals nearly closed in 2024(i.e., ~2 %), rates consistently remained lower at community hospitals over time. This likely reflects persistent structural differences between the two settings that could be further mitigated. Older male patients and those with federal insurance at community hospitals contributed most to these differences, suggesting that collecting PROMs from these populations might be even more challenging in community settings. While these populations are generally less likely to respond to PROMs [18,22,23], lower response rates at community hospitals may also stem from system- and provider-level factors. These may include leadership support, provider engagement, and proficiency in data collection [9,11–16,24,25]. Successful PROM implementation relies on the coordination across systems, providers, and patients. Addressing these system- and provider-level barriers, along with patient outreach, may help close the gap between community and academic hospitals effectively.

### Limitations

First, most patients were non-Hispanic White, limiting our ability to detect differences across racial/ethnic groups. Second, most patients were healthy with few comorbidities, thus also limiting our ability to detect differences by the degree of comorbidity. Third, the large sample size in this study might have contributed to the statistical significance of the findings. Therefore, caution is warranted when interpretating these results. Fourth, because 2020 was included as the first year of our trend analysis, the COVID-19 pandemic may have introduced barriers to PROM collection, resulting in relatively lower response rates compared with subsequent years. Finally, our findings may not be generalizable as we utilized data from a single health system.

## Conclusion

PROM collection significantly improved in radiation oncology clinics at MGB-affiliated community and academic hospitals over the four years of data analyzed. However, collection rates at community sites remained consistently lower by a small degree. Differences were attributable to males, old adults, and those with federal insurance. Our study highlights differences based on these known factors, but there are certainly unmeasured factors at work. The implementation of PROMs in clinical care requires continued vigilance at patient, provider, and system levels to foster adoption and sustainment.

## Supplementary Material

1

## Figures and Tables

**Table 1:** Comparisons of demographic and clinical characteristics of patients between academic and community sites, 2020–2024 (%)^†^.

	2020 (N = 8892)		2021 (N = 10,364)		2022 (N = 11,079)		2023 (N = 12,250)		2024 (N = 12,254)	
	Academic (N = 4678)	Community (N = 4214)	Academic (N = 5503)	Community (N = 4861)	Academic (N = 6012)	Community (N = 5067)	Academic (N = 6573)	Community (N = 5677)	Academic (N = 6412)	Community (N = 5842)
**Age**	***p* < .001**		***p* < .001**		***p* < .001**		***p* < .001**		***p* < .001**	
**Year (Median, IQR)**	66 (57 – 73)	68 (60 – 75)	67 (58 – 74)	68 (60 – 75)	67 (59 – 74)	69 (60 – 75)	67 (58 – 74)	69 (61 – 76)	67 (59 – 74)	69 (61 – 76)
**Age group**	***p* < .001**		***p* < .001**		***p* < .001**		***p* < .001**		***p* < .001**	
≤ 64	44	38	42	39	40	37	42	35	40	34
> 64	56	62	58	61	60	63	58	65	60	66
**Sex**	*p* = .12		***p* < .001**		***p* < .001**		***p* < .001**		*p* = .12	
Male	50	49	51	47	55	47	52	49	50	48
Female	50	51	49	53	45	53	48	51	50	52
**Race/ethnicity**	***p* < .001**		***p* < .001**		***p* < .001**		***p* < .001**		***p* < .001**	
Non-Hispanic White	80	81	80	81	79	80	79	80	78	79
Other	14	5	15	6	15	6	16	6	16	7
Unknown/declined	6	14	5	13	6	14	5	14	6	14
**Education**	***p* < .001**		***p* < .001**		***p* < .001**		***p* < .001**		***p* < .001**	
High school or less	24	27	23	26	22	25	22	25	22	26
College or above	70	47	71	49	71	50	70	50	70	50
Unknown/declined	6	26	6	25	7	25	8	25	8	24
**Insurance types** ^‡^	*p* = .07		*p* = .52		*p* = .73		*p* = .42		*p* = .25	
Federal	45	47	45	45	45	45	42	43	41	43
Commercial	55	53	55	55	55	55	58	57	59	57
**Employment**	***p* < .001**		***p* < .001**		***p* < .001**		***p* < .001**		***p* < .001**	
Employed	38	33	39	35	40	36	42	37	43	37
Unemployed	10	7	10	7	9	6	10	6	9	6
Retired	44	41	44	40	43	40	41	40	40	40
Student	0	0	0	0	0	0	0	0	1	0
Disabled	6	3	5	4	5	4	5	4	4	3
Unknown/declined	2	15	2	14	3	14	3	14	4	14
**Preferred language** ^‡^	***p* < .001**		***p* < .001**		***p* < .001**		***p* < .001**		***p* < .001**	
English	94	98	95	98	94	98	94	98	94	98
Non-English	6	2	5	2	6	2	6	2	6	2
**Comorbidity** ^‡^	***p* < .001**		***p* < .001**		***p* < .001**		***p* < .001**		***p* < .001**	
0,1	93	95	92	95	92	96	93	95	93	96
2 +	7	5	8	5	8	4	7	5	7	4
**Cancer types**	***p* < .001**		***p* < .001**		***p* < .001**		***p* < .001**		***p* < .001**	
Breast cancer	18	32	17	34	15	35	17	34	19	34
Prostate cancer	22	27	24	26	29	27	25	29	23	29
Lung cancer	14	12	13	12	12	11	11	10	11	10
Other	46	29	46	28	44	27	47	28	47	27
**Time since diagnosis**	***p*= .005**		***p* < .001**		***p* < .001**		***p*= .01**		***p*= .02**	
**Month (mean [SD])**	22 (18)	20 (16)	25 (22)	22 (18)	28 (25)	24 (20)	28 (27)	25 (22)	29 (29)	26 (24)

†: Except for age and time since diagnosis, (column) percentages were present at this table.

‡: Patients who were missing Charlson comorbidity index (5 %), insurance types (2 %), or preferred language (1 %) were excluded due to the small proportions.

Interpretation: We compared patients’ demographic and clinical characteristics between academic and community sites by year. For instance, in 2020, community sites had a higher proportion of patients aged 65 years old or older compared with academic sites (46 % vs. 62 %).

## Data Availability

The data underlying this article are subject to an embargo of 12 months from the project end date. Once the embargo expires, the data will be available in Harvard Dataverse.

## Appendix A. Supporting information

Supplementary data associated with this article can be found in the online version at doi:10.1016/j.apro.2025.100301.
