# Supplementary material for: Towards enhancing the representativeness of patient-reported outcomes collection at community cancer centers
Source: Adv Patient Rep Outcomes. Author manuscript; Available in PMC 2026 Aug 19. (PMC13484357; doi:10.1016/j.apro.2025.100301)
Supplement: Supplementary file 1 [file NIHMS2187164-supplement-1.docx]

**Supplemental Materials**

**Towards Enhancing the Representativeness of Patient-Reported Outcomes Collection at Community Cancer Centers**

Chengbo Zeng, PhD^1,2^, Neil E. Martin, MD, MPH^2,3^, Peter M. Meyers, BS^4^, Marc DeAngelo, MSM^4^, Tanja Xing-Yu He, MDc^1^, Yu-Jen Chen, MPH^5^, Boyu Ren, PhD^2,6^, Andrea L. Pusic, MD, MHS^1,2,7^, Maria O. Edelen, PhD^1,2,8^, Jason B. Liu, MD, MS^1,2,9^

**Author affiliations**

1. Patient Reported Outcomes, Value and Experience (PROVE) Center, Department of Surgery, Brigham and Women’s Hospital, Boston, Massachusetts, USA
2. Harvard Medical School, Boston, Massachusetts, USA
3. Department of Radiation Oncology, Brigham and Women’s Hospital and Dana-Farber Cancer Institute, Boston, Massachusetts, USA
4. Office of the Chief Medical Officer, Mass General Brigham, Boston, Massachusetts, USA
5. Center for Surgery and Public Health, Department of Surgery, Brigham and Women’s Hospital, Boston, Massachusetts, USA
6. McLean Hospital, Belmont, Massachusetts, USA
7. Division of Plastic and Reconstructive Surgery, Department of Surgery, Brigham and Women's Hospital, Boston, Massachusetts, USA
8. Behavioral and Policy Sciences, RAND Corporation, 20 Park Plaza #910, Boston, Massachusetts, USA
9. Division of Surgical Oncology, Department of Surgery, Brigham and Women's Hospital, Boston, Massachusetts, USA

**Figure S1.** Comparison of response rates between community and academic sites for the selected patient subgroups

**(a) Age > 64**

**(b) Males**

**(c) Other Racial/ethnic Groups^‡^**

**(d) Federal Insurance^‡^**

**(e) High School or Less^‡^**

**(f) CCI ≥ 2^‡^**

‡: patients who were missing race/ethnicity, insurance types, education level, or Charlson comorbidity index were excluded.

*: *p*-value in bold was statistically significant for the tests across demographic and clinical characteristics based on the Benjamini-Hochberg adjustment.

Interpretation: We examined between-site differences in response rates among older adults, males, racial/ethnic minorities, patients with federal insurance, high school or less education, and those with more comorbidities. For instance, in Figure S1.a, we found that there were consistently large differences in response rates between community and academic sites among older adults, from 2020 to 2024.
